# Experience in the use of non-pneumatic anti-shock garment (NASG) in the management of postpartum haemorrhage with hypovolemic shock in the Fundación Valle Del Lili, Cali, Colombia

**DOI:** 10.1186/s12978-017-0325-2

**Published:** 2017-05-12

**Authors:** María Fernanda Escobar, Carlos Eduardo Füchtner, Javier Andrés Carvajal, Albaro José Nieto, Adriana Messa, Sara Sofía Escobar, Angélica María Monroy, Angélica María Forero, José David Casallas, Marcela Granados, Suellen Miller

**Affiliations:** 1 Department of Gynecology and Obstetrics, Fundación Clínica Valle del Lili, Universidad ICESI, Cali, Colombia; 2 Department of Infertility, Reproductive Health Center, Universidad ICESI, Cali, Colombia; 3 Department of Gynecology and Obstetrics, Fellow of Intensive Care Unit, Fundación Clínica Valle del Lili, Cali, Colombia; 4 Department of Health Sciences, Medicine School, Universidad ICESI, Fundación Clínica Valle del Lili, Cali, Colombia; 5 Clinical Investigation Centre, Department of Gynecology and Obstetrics, Fundación Clínica Valle del Lili, Cali, Colombia; 6 Internal Medicine, Intensive Care Unit, Fundación Clínica Valle del Lili, Cali, Colombia; 7 Safe Motherhood Programs, Department of Obstetrics, Gynecology & reproductive Sciences, Bixby Center for Global Reproductive Health and Policy School of Medicine, University of California, San Francisco (UCSF), San Francisco, USA

**Keywords:** Postpartum haemorrhage, Hypovolemic shock, Emergency treatment, Gravity suits, Hemorragia postparto, Choque hipovolémico, Tratamiento de emergencia, Trajes gravitatorios

## Abstract

**Background:**

The aim of this case series is to describe the experience of using the non-pneumatic anti-shock garment (NASG) in the management of severe Postpartum hemorrhage (PPH) and shock, and the value of implementing this concept in high-complexity obstetric hospitals.

**Methods:**

Descriptive case series of 77 women that received NASG in the management of PPH with severe hypovolemic shock from June 2014 to December 2015. Vital signs, shock index (SI), the lactic acid value and the base deficit were compared before and after NASG application.

**Results:**

Fifty-six (77%) women had an SI > 1.1 at the time shock management was initiated; 96% had uterine atony. All women received standard does of uterotonics. The average time between the birth and NASG applications was 20 min. Forty-eight percent of women recovered haemodynamic variables in the first hour and 100% within the first 6 h; 100% had a SI < 1.0 in the first hour. The NASG was not removed until definitive control of bleeding was achieved, with an average time of use of 24 h. There were no mortalities.

**Conclusions:**

In this case series of women in severe shock, the NASG was an effective management device for the control of severe hypovolemic shock. It should be considered a first-line option for shock management.

## Resumen

**Fondo:**

El propósito de esta serie de casos es describir la experiencia de utilizar el traje antichoque no neumático (TANN) en el manejo de la hemorrágia postparto (HPP) y el choque severo, y el valor de implementar este concepto en hospitales obstétricos de alta complejidad.

**Métodos:**

Series de casos descriptiva de 77 mujeres que requirieron TANN en el manejo de HPP con choque hipovolémico severo desde junio del 2014 a diciembre del 2015. Se compararon los signos vitales, el índice de choque (IS), el valor de ácido láctico y el déficit de base antes y después de la colocación del TANN.

**Resultados:**

Cincuenta y seis (77%) mujeres tuvieron un IS > 1,1 en el momento en que se inició el tratamiento del choque; 96% tenían atonía uterina. Todas las mujeres recibieron una dosis estándar de uterotónicos. El tiempo promedio entre el nacimiento y la colocación del TANN fue de 20 minutos. Cuarenta y ocho por ciento de las mujeres recuperaron las variables hemodinámicas en la primera hora y el 100% en las primeras 6 horas; 100% tenían un IS <1,0 en la primera hora. El TANN no se retiró hasta que se alcanzó el control definitivo de la hemorragia, con un tiempo promedio de uso de 24 horas. No hubo muertes.

**Conclusiones:**

En esta serie de casos de mujeres en choque severo, el TANN fue un dispositivo de manejo efectivo para el control de choque hipovolémico grave. Debe considerarse una opción de primera línea para el manejo del choque.

## Plain English Summary

Postpartum hemorrhage (PPH) is the principal cause of maternal death and illness in Latin America. These women die as a result of heavy, uncontrolled bleeding. When excessive blood is lost, there is inadequate oxygen in the main organs; the brain, heart and lungs, and the woman goes into shock.

The non-pneumatic anti-shock garment (NASG) is a first line intervention for the management of women with severe PPH and hypovolemic shock. The NASG applies circumferential pressure to the lower part of the body, which increases the blood flow to the brain, heart and lungs. In this study we report on the experience of using the NASG in the management of PPH and the value of implementing the device in obstetric units where high risk patients are managed.

Seventy-seven patients with postpartum hemorrhage received the NASG. All women received standard doses of medications to contract the uterus. At 6 h all women had recovered their heart rate and blood pressure. Bleeding was controlled with the NASG rapidly, and 24 h later it was removed.

In conclusion, the NASG is a viable management alternative in cases of PPH and shock, especially as it is very easy to use across the different levels of care, from lower level facilities up to the high-complexity level.

## Background

The 2015 inter-agency report on maternal mortality established a global maternal mortality ratio (MMR) of 216 per 100 thousand live births. Ninety-nine percent of maternal deaths were concentrated in developing countries and hemorrhage was the principal cause of maternal mortality and morbidity in Latin America [1–4]. In 2015 in Colombia, where this study was conducted, the MMR was 52 per 100 thousand live births [5], approximately 50% of deaths were due to hemorrhage; the majority of hemorrhage was due to postpartum hemorrhage (PPH) [6].

Mortality due to PPH is directly related to the duration and the amount of bleeding [7]. With a loss of blood volume of 40%, hypovolemic shock, multiple organ dysfunction and global hypoxia develop, with severe metabolic damage. Shock becomes irreversible when hypothermia, coagulopathy, and metabolic acidosis (triad of death) are present [8].

The non-pneumatic anti-shock garment (NASG) is a first-line intervention for the management and stabilization of women with PPH in hypovolemic shock. The suit applies between 20 and 40 mmHg of circumferential pressure to the lower part of the body, which increases the venous flow and the cardiac output allowing a systematic distribution of flow to vital organs [9]. The direct compression of the descending aorta counteracts the bleeding from the uterine arteries and the vasculature of the mesenteric bed [10].

Different comparative studies have shown a reduction of approximately 50% in maternal mortality due to PPH following the introduction of the NASG at the tertiary level [11–15]. A systematic review of five of these studies (*n* = 2330) showed a significant reduction in mortality, loss of blood, and a more rapid recovery of shock index (SI) in women when NASG was used [16]. A randomized clinical trial assessing the rapid implementation of NASG demonstrated a statistically significant reduction in SI recovery. These results led to the introduction of the NASG in the World Health Organization’s (WHO) 2012 management guide for the management of the PPH [17] and its inclusion within the 2013 policies for health and education for medical personnel. The International Federation of Gynaecology and Obstetrics (FIGO) has recommended that NASGs be used for clinical stabilization and transport of all women with PPH from lower to higher levels of care [18].

The Fundación Valle del Lili (FVL) is a private non-profit medical centre with emphasis on the maternity care of women with complications in the High Complexity Unit (UACO), which corresponds to an obstetric critical care unit. The objective of this article is to report the outcomes obtained after the introduction of the NASG in the management of women with massive PPH, hypovolemic shock, and hypoperfusion in UACO.

## Methods

This case series was conducted between June 2014 and December 2015, in the UACO. The primary data sources were the clinical records in the patient’s file. Data were collected using a tool specifically designed to collect the variables of interest. Demographic variables were collected, as were variables related to the hemorrhage etiology, management, and outcomes for the patient cohort.

We developed the research protocol according to requirements of the Clinical Investigations Centre at the FVL. The Institutional Ethics Committee at FVL approved the protocol. All women and their families were informed of the need to use NASG, and were also informed regarding the technical, health, and safety aspects of the device.

The case series included all the pregnant women who presented with PPH (loss of more than 500 or 1000 milliliters (mL) after vaginal delivery or Caesarean section, respectively), with hemodynamic instability (hypovolemic shock), and clinical indicators confirming hypoperfusion. We used the NASG to control bleeding in conjunction with the institutional clinical protocol for the management of PPH. The diagnosis of hypovolemic shock was classified using Baskett’s criteria [19].

In all women, Shock Index (SI), the ratio between the heart rate and the systolic blood pressure, was measured at the time of diagnosis. SI has been studied in the obstetric population as a valuable marker of hemodynamic instability in cases of massive PPH, and has been found to be directly related to the probability of massive transfusion and the development of coagulopathy [20, 21].

The variables hypoperfusion, lactic acid and base deficit (mmol/L) were measured at the time of diagnosis of PPH and at 24 h after diagnosis [22]. The institutional clinical protocols in the intensive care, high complexity units of the FVL requires measurement of the Acute Physiology and Chronic Health Evaluation II (APACHE II) score for all women in order to evaluate and classify the acuity of their critical state. In this series, the score was established for all women in the first 24 h after the haemorrhage. Recovery from hypovolemic shock and hypoperfusion is assumed in women with an SI of ≤ 1, with lactic acid < 2 mmol/L and/or a base deficit < −6 mmol/L. We excluded women who were admitted to the institution with an NASG from other institutions, with haemodynamic alterations due to another condition, with haemorrhage prior to delivery due to an obstetric aetiology, and those with previously documented coagulation disorders.

The standard medical protocol for PPH treatment included uterotonic medications. The first choice was Oxytocin with doses from 80 to 160 mile-units (mU) per minute via drip with an infusion pump. Methylergometrine was the second-choice drug in doses of 0.2 milligrams (mg), intramuscularly, at the beginning of the treatment, followed by a second dose at 20 min and then every 4 h, up to a total of five doses (Methylergometrine was used only if the patient was not hypertensive and had no contraindications for its usage). The third choice was Misoprostol with a dose of 800 micrograms (mcg) sublingually, or intra-rectally, where sub-lingual delivery was not possible.

The data analysis was carried out using STATA®. The quantitave comparison between status at time of NASG application and recovery was performed using non-parametric methods (median and quartiles) and the categorical variables were analyzed using a distribution of frequencies. The APACHE II was divided into two levels, taking into account the median of the distribution.

## Results

A cohort of 77 women was identified, with postpartum hemorrhage and hypovolemic shock, hypoperfusion, all of whom received the NASG. This cohort corresponded to 33.9% of the total of pregnant women with PPH who were treated at the UACO during the study period. The workload of the High Complexity Obstetric Care unit for 2015 was 2104 deliveries. The main characteristics of the women are described in Table 1.

**Table 1:** Patient characteristics

Characteristics	Total number of women: 77
Age^a^	25 ± 6 Minimum and maximun (14–43)
Gestational stage at admission^b^	38 (34–39) Gestational stage minimum-maximum (20–41)
Antenatal care visits	70 (90%)
	Number of patients
● 1–2 visits ● 3–5 visits ● >5 visits	10 (14%) 29 (42%) 31 (44%)
Level of education	
● None ● Primary ● Secondary ● University	6 (8%) 7 (9%) 36 (47%) 28 (36%)
Parity at time of admission	
● Primiparous ● Second pregnancy ● Third pregnancy ● > Three pregnancies	40 (52%) 27 (35%) 6 (8%) 4 (5%)
With no previous complications	58 (75%)
Vaginal delivery	53 (69%)
Induced delivery	10 (13%)
Days hospitalized^b^	4 (3–7)
Days hospitalized in the ICU^b^	3 (2–3)

^a^Mean (SD: Standard Deviation)

^b^Median (IQR: Interquartile Range)

The main diagnosis at admission was severe preeclampsia (32%), followed by premature rupture of membranes (5%). The perinatal outcome was live birth in 88% of cases, with a median birth weight of 3137 g IQR (2555–3560 g). There were six cases (7%) with multiple pregnancies and nine stillbirths (three cases with early-onset severe preeclampsia, two cases with placental abruptio and 4 without indeterminate etiology). The principal hemorrhage aetiology was uterine atony (96%), 15% had retained placental tissue, and 5% had Placenta Previa. Six percent of women experienced a grade III vaginal tear at the time of delivery.

Seven (9%) patients with PPH were transferred from other hospitals. Among the women who started hemorrhage in our setting, the median time between the birth and NASG placement (placed at the time of recognition of hypovolemic shock) was 20 min IQR (10–42 min). Fifty-four women (70%) received an intrauterine tamponade with a Bakri balloon for 24 h, and eight (10%) were taken for B-lynch surgery. The NASG remained on these women. Two women with severe preeclampsia presented exsanguinating haemorrhage, which required a total abdominal hysterectomy (TAH) and damage control surgery. The NASG was not removed until definitive control of bleeding and hemodynamic stabilited was achieved. The majority of women had the NASGs removed by 24 h. Once removed, there was no need to reuse it in any patient.

Visual bleeding was estimated to be greater than 1000 mL in all women at the time the NASG was placed. The summary of the measurement of hypoperfusion and shock variables is shown in Table 2. Fifty-six women (73%) presented with a shock index ≥ 1.1 at the initiation of NASG.

**Table 2:** Comparison of Diagnostic variables for shock and hypoperfusion in women with PPH at time of NASG placement and removal

Shock variable	Number of Women: 77	
	When NASG applied	When NASG removed
Shock index (%)		
● 0.5–0.9 ● 1–1.5 ● >1.5	0 62 (81%) 15 (19%)	75 (97%) 2 (3%) 0
Lactic acid value^b^	3.1 ± 1.8 mmol/L Minimum and maximum values (0.9–12) mmol/	1.3 ± 0.6 mmol/L Minimum and maximum values (0,7–4.1) mmol/L
Mean difference in lactic acid	1.8 (±1.8) mmol/L	
Base deficit^a^	−7.4 ± 3.6 mmol/L Minimum and maximum values (−1.7–22) mmol/L	−4.2 ± 1.9 mmol/L Minimum and maximum values (−0.5/–8.5) mmol/L
Haemoglobin 24 h after PPH^b^	9.5 ± 1.8 g/dL Minimum and maximum values (5.2–14.1) g/dL	
APACHE (1st hour)^b^	8 (6–11) Minimum and maximum values (2–21)	

^a^Mean (SD: Standard Deviation)

^b^Median (IQR: Interquartile Range)

**Fig. 1:**
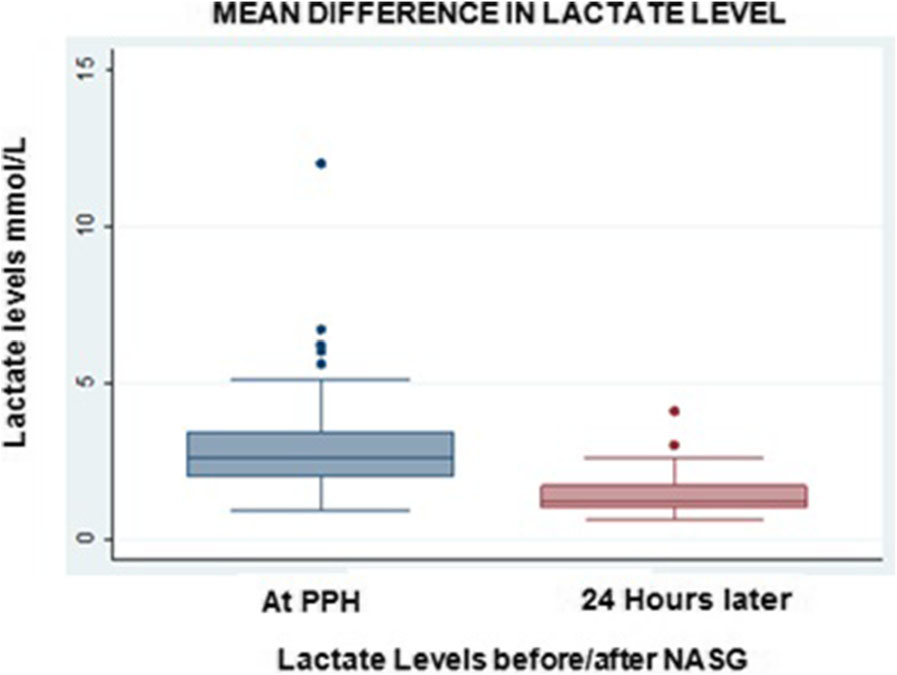
Mean Difference in lactate Level between Before/After NASG placement

Plotting individual lactate paired values before and after NASG placement with interval time of 24 h is showed in Fig. 1

100% of the women recovered normal heart rate and systolic blood pressure in the first 6 h of NASG use, 48% in the first hour, 100% of women presented an SI < 1 after the first hour of usage, shown in Fig. 1.

**Fig. 2:**
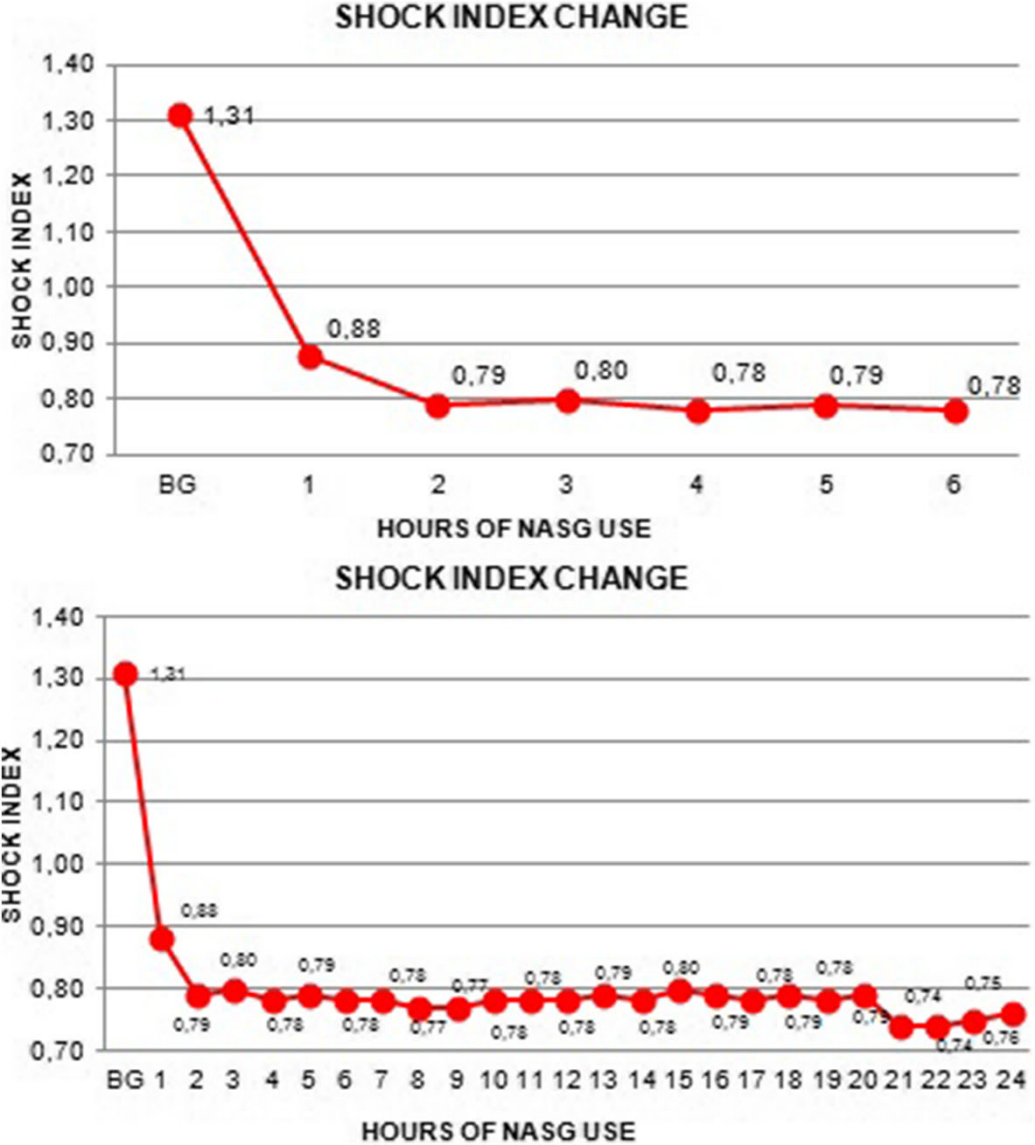
Shock Index Changes during the first 6 h and at 24 h of using the NASG. (BG: Before Garment)

**Fig. 3:**
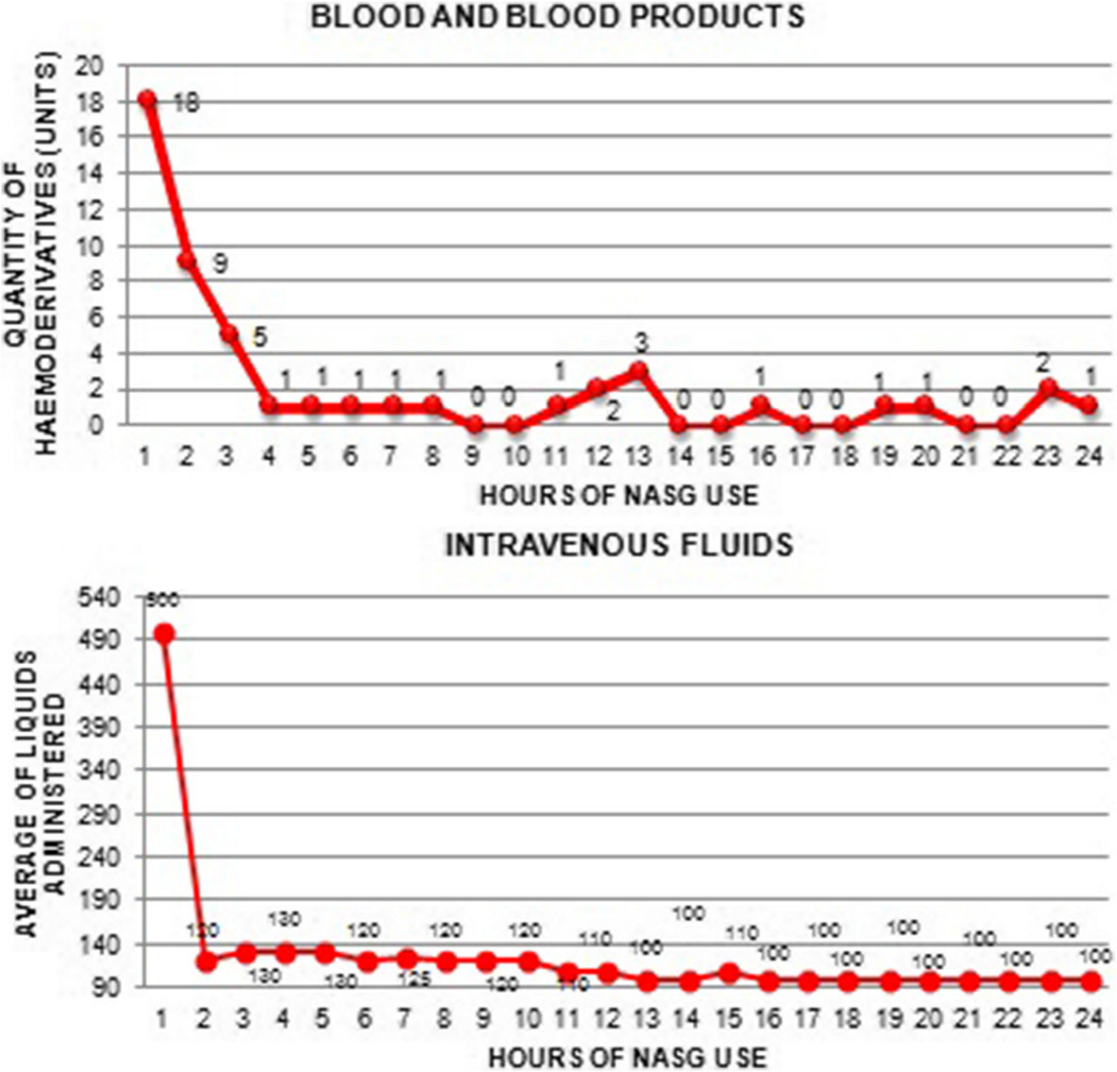
Administration of blood products and intravenous fluids with the NASG during the 24-h period

40.8% of the women received a transfusion of blood products. The administrations of blood products and intravenous fluids during the 24 h of NASG usage are described in Figs. 2 and 3.

Two women who received haemostatic surgery showed a level of lactic acid of 12 mmol/L, a base deficit of −22 mmol/L and an APACHE II score of 15. These two women recovered their vital signs following 24 h of management (0.8 mmol/L and −6.4 mmol/L, lactic acid and the base deficit values, respectively). No patient presented any infectious complications and no maternal deaths occurred in this cohort.

## Discussion

In this cohort of 77 women with hypovolemic shock and hypoperfusion secondary to PPH; 100% had an SI > 1.1 at the time NASG application and 100% had SI of < 1.0 in the first hour after placement of the NASG, with no mortality.

The NASG device was introduced to the UACO in July 2014 with full approval by the medical staff, after a comprehensive review of the available evidence [8–18]. Despite a gradual increase in the number of women presenting with PPH since the introduction of the NASG, there has been a progressive decrease in the percentage of women with TAH and damage control surgery, which may be associated with an improvement in the management strategies that included the use of NASG.

This case series of the NASG as part of the management of women with hypovolemic shock due to PPH, is among the first in which the magnitude of haemodynamic impairment was objectively corroborated with SI, lactic acid concentrations and the base deficit measurement at the time of the placement of the NASG and at 24 h after placement. The SI is reportedly the most accurate clinical parameter to determine blood loss by PPH. When the value is ≥ 1.0, it is directly related with haemodynamic instability in massive PPH; if it is > 1.1, it will (in 89% of all cases) require massive transfusion [19–21].

The effect of the swift application of NASG on the resuscitation of women with PPH has been previously reported, showing that when this process occurs in the first 2 h after the haemorrhagic event, the risk of death is reduced by 57% [23]. The average time for NASG implementation in this cohort was 20 min and, in all cases, it was applied in less than 1 h. This may be associated with the rapid clinical recovery in SI, where 100% of the women presented an SI of < 1.0 after the first hour of NASG use.

The successful NASG use in conjunction with Bakri balloons in a severely ill patient with PPH has been described on only one occasion [24]. In our cohort, 54 women (70% of the total), received a Bakri balloon and an NASG as a “sandwich” approach; all women had a rapid recovery. These outcomes may be secondary to the combination of the effects of the NASG restoring the circulatory volume while decreasing blood flow through the external compression of blood vessels and to the internal pressure of the Bakri ballon against the inside of the uterus (Bakri balloon). This combination is a relatively low-cost, non-surgical, intervention that allows a very close monitoring of uterine bleeding with good effectiveness [25]. There was no evidence of any differences in the recovery time, or of the decrease in SI, in the women who received both therapies (NASG or NASG + Bakri Balloon).

Our findings were similar to other studies that reported the successful use of NASG in women who also received B-lynch surgery, hysterectomy or damage control surgery [12]. In our cohort, eight women received hemostatic surgery and two women received a hysterectomy and damage control surgery. In no case was the NASG removed during surgery. The timing of removal of the NASG was based on the traditional indications and traditional perfusion variables indicating hemodynamic stability; no replacement was necessary after removal in any case.

Based on the literature [26], there was a 90% probability of the need for massive transfusion for the women in this series who had an SI ≥ 1.1 (72% (*n* = 54) of the total sample). Despite the magnitude of the bleeding, only 40% of the women required a transfusion of blood products and these were administered in the majority of cases in the first hour of management; 37% (*n* = 29) women received a transfusion of red blood cells, of whom 33% (*n* = 25) received two units. Only three women (3.8%) received a massive transfusion with red blood cells, platelets, plasma and cryoprecipitate. This result has important implications for low-resourced settings where blood availability is low.

The use of NASG in severely compromised women, alongside the management protocol described by the UACO, led to recovery from hypoperfusion and achieved the resuscitation goals in all women in the first 24 h; the majority of women recovered in the first hour after NASG application. Although this series is not intended to demonstrate the unique effect of NASG in these results, its incorporation in the management of these severely ill women enabled the control of bleeding and the swift recovery of cellular oxygenation, thus avoiding organ dysfunction and death.

In the present cohort, no adverse events or major complications were found. The median value for the APACHE II score in this cohort was 8, with an attributable expected mortality of 10% in these women; as has been seen, there was no case of maternal mortality. Future research should compare baseline or pre-intervention outcomes of the same variables with NASG implementation.

## Conclusions

The success of the NASG, in conjunction with medical and surgical strategies for the management of women with PPH and hypoperfusion, suggests that this device should become part of the protocol for handling hypovolemic shock secondary to severe hemorrhage in the labour units of high-complexity hospitals. Its use may decrease the probability of more invasive treatments, higher rates and volumes of blood transfusions, and costs. Even in women who required rescue strategies to control bleeding, the concomitant use of the NASG was not associated with any major complications, and could be associated with better outcomes in women with an 80%, or higher, likelihood of dying at the time of the placement of the NASG.

In this study the NASG was an aid to rapid restoration of circulatory normality, and helped avoid metabolic derangement and service burdens, such as need for massive transfusion or surgery. The variety of clinical experiences reported with the use of NASG suggests its effectiveness accross a wide range of settings and resources. Based on the present report, we believe that the NASG is a viable tool for managing women with hypovolemic shock secondary to severe PPH at the highest referral level. It is easy to learn how to apply, there are no safety issues, and it can be used across different levels of care, from the smallest primary health care unit up to the high-complexity level.
